# Anaphylactoid reaction caused by sodium ceftriaxone in two horses experimentally infected by *Borrelia burgdorferi*

**DOI:** 10.1186/s12917-015-0478-6

**Published:** 2015-08-12

**Authors:** Roberta Carvalho Basile, Gabriela Gomes Rivera, Lara Antoniassi Del Rio, Talissa Camargo Mantovani de Bonis, Gabriel Paiva Domingues do Amaral, Edson Giangrecco, Guilherme Ferraz, Natalino Hajime Yoshinari, Paulo Aléscio Canola, Antonio Queiroz Neto

**Affiliations:** Faculdade de Ciências Agrárias e Veterinárias, UNESP – Univ Estadual Paulista, Campus Jaboticabal, Departamento de Morfologia e Fisiologia Animal, Laboratório de Farmacologia e Fisiologia do Exercício Equino (LAFEQ), Jaboticabal, SP 14884-900 Brazil; Departamento de Clínica e Cirurgia Veterinária, Jaboticabal, Brazil; Faculdade de Medicina da Universidade de São Paulo FMUSP, Departamento de Reumatologia, São Paulo, Brazil

**Keywords:** Hypersensitivity, Lyme, Colic, Laminitis

## Abstract

**Background:**

Lyme borreliosis is a disease transmitted by ticks to mammals, especially in horses and humans. Caused by a spirochete *Borrelia burgdorferi*, it can result in lameness, arthritis, carditis, dermatitis and neurological signs. Anaphylactoid reactions are severe responses caused by direct action of substances (drugs, toxins), which can pose risks to life. Still poorly documented in horses, these reactions are caused by the effects of inflammatory mediators such as histamine, kinins and arachidonic acid metabolites. The last two are the most clinically relevant for the species.

**Case presentation:**

The simultaneous occurrence of anaphylactoid reaction in two horses experimentally infected by *Borrelia burgdorferi* undergoing intravenous treatment with ceftriaxone sodium is reported. It was administered 4.7 × 10^8^ spirochetes intradermal and subcutaneous applications in both horses to evaluate clinical aspects of the Lyme disease, 95 days before the application of sodium ceftriaxone. During the administration, one horse (a gelding) showed immediate and severe anaphylactoid symptoms such as urticaria, dyspnea, tachycardia, and eyelid edema, which were controlled by injecting dexamethasone. After 1 day, it expressed signs of abdominal discomfort, caused by severe bloat, which was treated surgically via celiotomy. Subsequently, this gelding had piroplasmosis and severe anemia, requiring treatment with an antimicrobial and blood transfusion. Second horse (a mare) showed signs of hypotension during the application of the antibiotic, which disappeared only when the application was interrupted. Days after the event, the mare developed moderate large colon bloat, which was treated with medication only. Subsequently the mare was evolved into the prodromal phase of laminitis in one of the forelimbs, which was treated for 10 days with non-steroidal anti-inflammatory and rheology modifying drugs and cryotherapy.

**Conclusions:**

From the two cases presented here, it does appear that sodium ceftriaxone can induce anaphylactoid reactions in horses infected by *Borrelia burgdorferi*, which may evolve into colic syndrome, laminitis and the occurrence of opportunistic infections. However, further evidence should be collected in order to draw definite conclusions.

## Background

Lyme borreliosis is a multisystemic disease that affects humans, domestic and wild animals, transmitted by ticks and caused by spirochete *Borrelia burgdorferi* sensu lato. In horses, were necessary at least 18 h of tick attachment to transmission of the bacteria to the host, which can present lameness, arthritis, uveitis, encephalitis, abortion, foal mortality and recurrent hemoparasites infections. The treatment can be conducted using tetracycline, doxycycline or ceftiofur [1]. Conducting sodium ceftriaxone therapy for borreliosis has been reported only in humans [2]. Anaphylaxis is the most severe form of allergic reaction called Type 1 hypersensitivity, and occurs in horses mainly in response to toxins from venomous animals, drugs and food allergens [3]. The classical anaphylactic reaction is caused by the IgE antibodies binding onto the surface of mast cells and basophils with subsequent release of inflammatory mediators [3–6]. Anaphylactoid reactions are symptomatically similar, but they are caused by direct action of toxic substances (e.g. endotoxins, certain drugs and chemicals) with the subject has not necessarily been previously “sensitized” [5]. They do not involve IgE participation and occur through a direct nonimmune-mediated release of the same mediators [7]. The main mediators of a reaction are biogenic amines (histamine, serotonin, catecholamines), vasoactive polypeptides (kinins, cationic proteins, complement system anaphylaxins C_3A_, C_5A_, C_567_), lysosomal enzymes, vasoactive lipids (prostaglandins, endoperoxides, thromboxanes), phospholipids (platelet activating factor) and chemotactic substances [5, 6]. Anaphylactoid reactions are difficult to be reproduced experimentally, however there are reports of anaphylaxis induction experiments in horses by sensitization with bovine serum [8] that were conducted on 20 adult ponies under anesthesia. Cardiovascular, respiratory, hematological, electrolyte and biochemical responses were evaluated during the acute phase. The results contributed significantly to describing the cascade of events that occur during anaphylaxis in the species. The authors reported that the first event occurred about 2 min after contact with the antigen. Blood pressure (common carotid artery) decreased significantly while pulmonary artery pressure increased and the pressure of the abdominal vena cava decreased slightly. During anaphylaxis, there was hemoconcentration, leukopenia, thrombocytopenia, and hyperkalemia. Initially, plasma concentrations of histamine and bradykinin increased significantly (more than four times). Clinically, tachypnea and tachycardia were also observed, which returned to normal in about 12 min. The therapeutic response of 16 ponies with induced anaphylaxis to antagonistic drugs of the main anaphylactic mediators [9] has also been evaluated. The author compared the antagonist effects of burimamide and tripelennamine on histamine and also evaluated methysergide as serotonin blocker. The efficacy of meclofenamate sodium, and acetylsalicylic acid as non steroidal anti-inflammatory drugs was evaluated, as well. The results show that meclofenamate sodium and acetylsalicylic acid effectively inhibited and managed the anaphylactic cardiovascular and respiratory symptoms, suggesting that in horses, prostaglandins, thromboxanes and kinins have greater clinical relevance to immediate hypersensitivity [6]. On the other hand, histamine is primarily responsible for most adverse events in humans and dogs [4]. There are few case reports in the literature about anaphylaxis and anaphylactoid reactions in horses. There is a case description of a gelding that received intravenous ivermectin and developed a lethal anaphylactic reaction [10]. More recently, there is an unusual case of anaphylaxis in a neonatal foal caused by inadvertent intravenous administration of breast milk [11], which was reversed with epinephrine, and dexamethasone, and intensive outpatient treatment for 9 days. The most recent publications on IgE-mediated hypersensitivity in horses emphasize only dermatological aspects as signs of atopy, allergy to insect stings, food and parasitism associated allergies [12, 13]. Sodium Ceftriaxone, classified as third-generation cephalosporin, is a broad spectrum antibiotic with a beta lactam ring in its structure and capable of bypassing the blood-brain barrier [14]. Its pharmacokinetics has been evaluated in mares [15], adult horses [16] and foals [17]. These studies were performed by intravenously injecting a single dose between 25 and 50 mg/kg, diluted in saline solution. No adverse drug reactions have been reported in these studies. To date, there are no reports regarding the occurrence of adverse reactions to ceftriaxone sodium in horses, as has already been observed in humans [16]. Therefore, this case report describes the occurrence of non-fatal anaphylactic reaction in two horses after intravenous administration of ceftriaxone sodium, the immediate clinical outcome, consequences and subsequent therapeutic strategies adopted.

## Case presentation

Two sound horses, a gelding and a mare chosen from the group of experimental horses of the University, approximately eight years old, were experimentally infected with *Borrelia burgdorferi*, using a dose of 4.7 × 10^8^ bacteria per horse, divided in four simultaneous applications (2 subcutaneous and 2 intradermal), in the region of the superficial cervical lymph nodes, with the approval of the Ethics Committee on Animal Use - CEUA of UNESP (protocol 001968/13). The objective of the experiment was to compare the clinical and laboratorial differences between the infection of *Borrelia burgdorferi* strain G39/40 in horses versus the Borreliosis diagnosed in Brazil in addition to their treatment viability with sodium ceftriaxone, since it is an effective antibiotic to control borreliosis, including its neurological forms [13]. There were no previous reports of sodium ceftriaxone adverse reactions in horses. Both horses had only mild acute clinical alterations in the first 14 days after infection, characterized by augmented submandibular lymph nodes, pale mucosa, dorsal sensibility and hyporexia. Ninety five days after experimental infection, both animals were injected intravenously (4 drops/s) ceftriaxone sodium at the dose of 25 mg/kg, diluted in 500 ml of sterile 0.9 % sodium chloride saline solution, by catheterization^1^^,^^2^ of the left jugular vein. The clinical and hematological parameters of both horses were checked before administration and were within normal range. However, the clinical and hematological parameters changed approximately 2 min after the drug was administered. Both horses that received sodium ceftriaxone developed an anaphylactoid reaction. The entire experiment was conducted on only these two animals.

### Case 1: Gelding

**Fig. 1:**
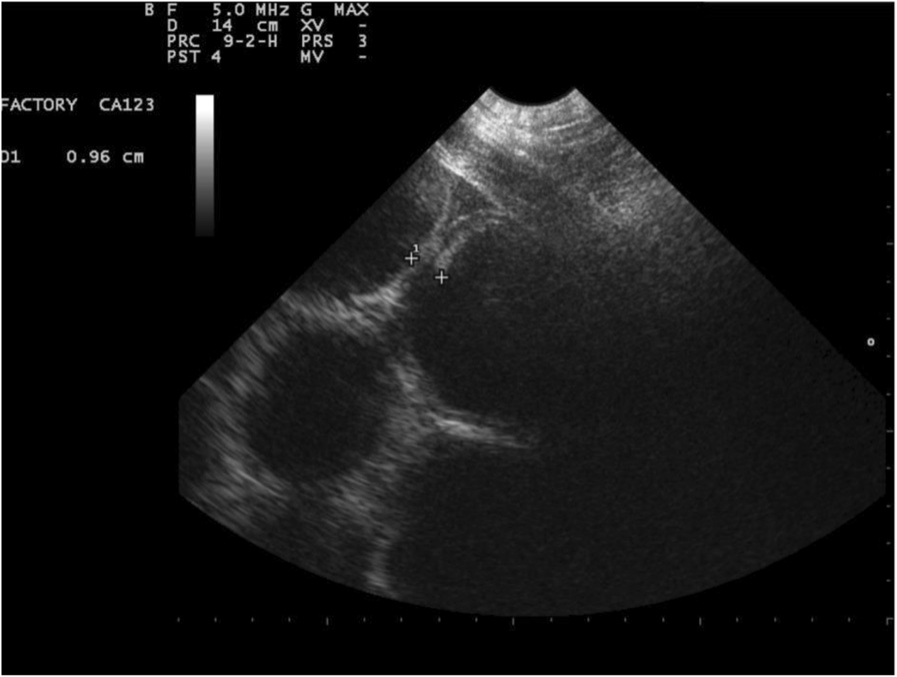
Abdominal ultrasound of the gelding with tympanic colic as a result of anaphylactic reaction to sodium ceftriaxone. Image obtained by ventral-abdominal positioning of the probe, caudal to xiphoid. Note the thickening of intestinal wall, distention, atony and *ileus* in segments of the small intestine. Diagnostic Imaging Sector - FCAV - UNESP - Jaboticabal

**Fig. 2:**
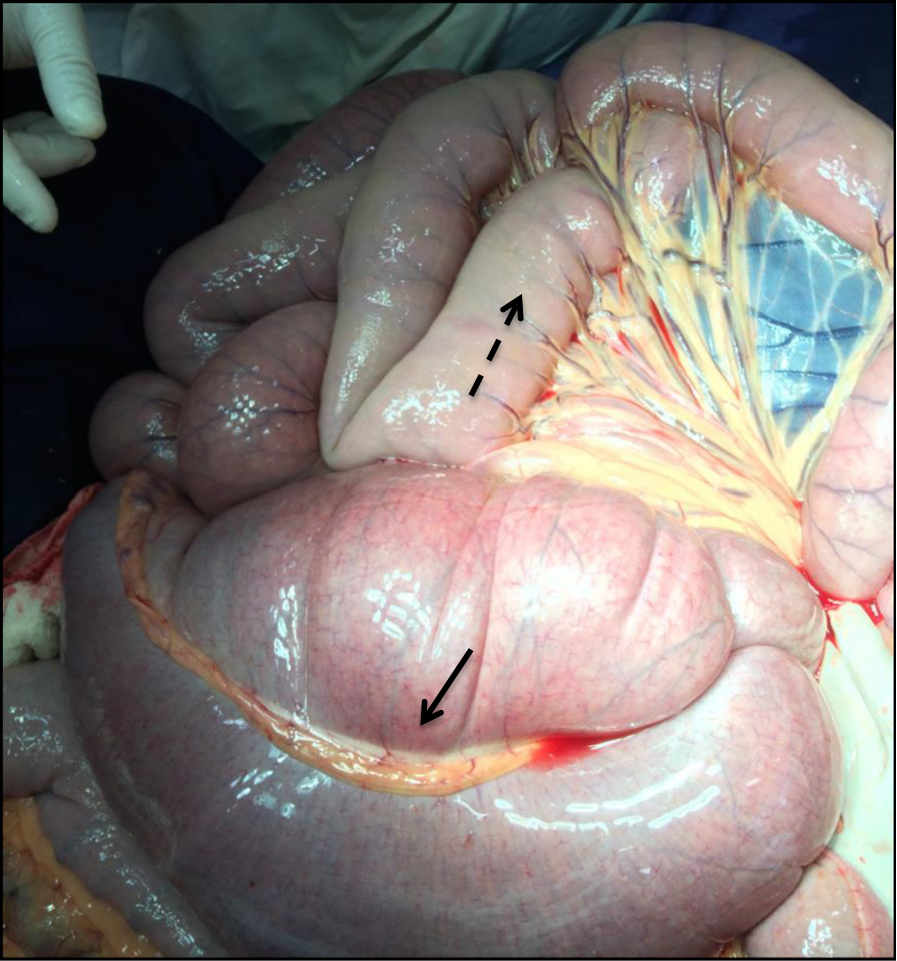
Exposure of the gelding cecum and jejunum during exploratory celiotomy. Full line arrow: cecum. Dashed line arrow: jejunal serosa showing edema due to anaphylaxis caused by sodium ceftriaxone, in lighter color compared to other intestinal loops

A male purebred Arabian horse weighing 410 kg, submitted to 6 h of food fasting had a heart rate (HR) of 40 bpm, respiratory rate (RR) of 22 mrm, rosy mucous membranes, capillary refill time (CRT) of 2 s, rectal temperature (RT) of 37.1 °C and hematological parameters within the reference range for the species (Table 1, Basal) before the administration of the antibiotic. About 17 min into the administration of ceftriaxone sodium (Day 1), after 205 mL of the solution had dripped, the horse showed tachycardia (heart rate = 96 bpm), dyspnea and urticaria on the neck left side (the same side where the drug was being injected). The drug dripping was discontinued immediately, but the animal still presented severe dyspnea. The gelding respiratory function normalized a minute after 0.05 mg/kg dexamethasone was injected. A noticeable swelling of eyelids was observed an hour after the allergic event. At this time the patient had a HR of 48 bpm, RR of 20 mrm, pale mucosa, RT of 37.7 °C and a slight reduction of intestinal motility. The horse behavior remained apathetic for the rest of the day, with no significant changes upon physical examination and was fed only hay. On day 2 in the morning, the gelding was fed 1.0 kg of commercial feed, but due to abdominal discomfort it positioned itself on lateral recumbency. Upon examination, the horse had severe bilateral abdominal distention, a HR of 37 bpm, RR 20 mrm, RT 37.2 °C, pale mucosa, intestinal hypomotility and yellowish and turbid peritoneal fluid. In the laboratory, the peritoneal fluid was characterized by total protein of 96 g/dL; pH 7.6; density 1.010 g/dL and 1250 cells/ml (71 % segmented neutrophils, 22 % lymphocytes and 7 % macrophages). Hematological and blood gas evaluation of venous blood showed anemia and hemoconcentration (Table 1, Day 2), metabolic and respiratory acidosis, hyponatremia, hypokalemia, hypochloremia, hyperglycemia, and a blood lactate concentration (Table 2, Day 2). The abdominal ultrasonography showed severe gaseous distension, *ileus*, along with wall thickening of small intestine (Fig. 1). The ultrasonography results were consistent with volvulus of the small intestine; therefore, the gelding was referred to the surgical center for exploratory celitomy. Before the surgery, an intravenous antibiotic therapy with 20.000U/kg potassium penicillin^3^ was initiated. During surgery, were observed gas distention throughout the small bowel, wall edema of the small and large colon, cecum, jejunum and *ileus* (Fig. 2). There were also retroflexion of the pelvic flexure and displacement of cecum. The small intestine milking was followed by gas aspiration of the cecum, colon repositioning and washing of the abdominal cavity with neomycin diluted in 10 % Sodium lactate Ringer's solution. After surgery, the horse recovered uneventful from anesthesia and defecated upon rising. The antibiotic therapy was continued post operatively with penicillin potassium (20,000U/kg), every 8 h, for three consecutive days. The patient was also treated with a single dose of both furosemide (0.5 mg/kg) and dexamethasone (0.05 mg/kg) to reduce the intestinal edema; 30 mL of Potassium Chloride (150 mg/mL) diluted in 1 L of Sodium Lactate Ringer's solution to correct the hypokalemia; 3 l of solution of 10 % DMSO solution (100 mL/L in Ringer's lactate solution), once daily for 2 days; and, flunixin meglumine (0.25 mg/kg) once daily for 3 days. The abdominal incision was cleaned using aqueous chlorhexidine solution while repellent was applied around the surgical wound twice a day, for 10 days. This patient progressed steadily until the eighteenth day after anaphylaxis, when it showed apathy, loss of appetite, fever of 40.1 °C, pale mucosa, 3 s CRT, HR 72 bpm, RR 44 mrm and severe back pain. A blood sample was collected using EDTA tube and sent to the Parasitology Laboratory for Polymerase Chain Reaction (Nested-PCR) to detect the presence of *Babesia caballi* and *Theileria equi*, as well as Real-Time PCR for *Anaplasma phagocytophilum*. The test revealed *Theileria equi* infection. Therefore, intramuscular treatment with imidocarb^4^ (4.0 mg/kg) was instituted, divided into two daily doses for 2 days, along with Dipyrone (25 mg/kg), every 12 h for 2 days. Subsequent to treating piroplasmosis, the gelding also revealed high serum concentrations of indirect bilirubin, creatine kinase, gamma glutamyl transferase, globulin and fibrinogen (Table 3, Day 20). On the twentieth day after anaphylactoid reaction, the gelding showed marked hypochromic normocytic anemia and neutrophilia (Table 1, day 20) and the patient was transfused with 6 L of whole blood. The following day, the blood parameters improved (Tables 1 and 3, Day 21). However, the horse showed signs of abdominal discomfort though sternal recumbence and anorexia with normal intestinal motility, fecal output and consistency, mild tachycardia and depression, suggestive of gastritis. A twenty-day course of omeprazole^5^ was instituted and a satisfactory improvement of its clinical and hematological status occurred 28 days after the anaphylaxis (Tables 1, 2 and 3, Day 28).

**Table 1:** Gelding red blood cell count before and after anaphylactic reaction to sodium ceftriaxone, which occurred on day 1

	Basal	Day 2	Day 3	Day 6	Day 10	Day 20	Day 21	Day 28	Reference^a^
Erythrocytes x 10^6^ [cells/ml]	8.40	6.72	6.10	6.32	6.30	*3.10*	6.20	6.50	6.0–9.7
Hemoglobin [g/dL]	12.5	10.6	9.8	9.8	9.2	*4.2*	8.6	10.0	8.3–14.4
Hematocrit [%]	38.0	*29.5*	*26.7*	*27.4*	*28.0*	*13.0*	*27.0*	30.0	30–44
MCV [fL]	45.2	-	-	-	44.4	41.9	43.5	46.2	36–52.1
MCH [pg]	14.9	-	-	-	14.6	13.5	13.9	15.4	11.5–18.2
MCHC [g/dL]	32.9	-	-	-	32.9	32.3	31.9	33.4	31.2–34.9
Leucocytes [cells/uL]	10100	9800	12900	5700	*11200*	*10800*	*14800*	9500	6400–10600
Basophiles [cells/uL]	0	0	0	0	0	0	0	0	0
Eosinophils [cells/uL]	808	0	0	100	112	216	148	285	0–320
Segmented Neutrophils [cells/uL]	6060	*7800*	*9100*	6600	8736	6804	*11692*	5700	2775–7530
Rod Neutrophils [cells/uL]	202	0	100	0	*448*	216	*1480*	285	94–420
Lymphocytes [cells/uL]	2727	2100	*800*	2900	1680	3348	1184	3135	1088–5096
Monocytes [cells/uL]	303	100	0	*400*	224	216	296	190	90–318
Platelets [cells/uL]	194000	246000	242000	279000	*323000*	90000	96000	*368000*	90000–322000

*MCV* mean corpuscular volume; *MCH* mean corpuscular hemoglobin; *MCHC* mean corpuscular hemoglobin concentration

^a^Reference ranges obtained for total blood cell count for 33 domestic horses of different breeds, serum negative for *Borrelia burgdorferi*, kept under the same feeding and management conditions

**Table 2:** Gelding blood gas analysis after anaphylactic reaction to sodium ceftriaxone, which occurred on day 1

	Day 2	Day 3	Day 28	Reference^a^
pH	*7.271*	*7.467*	7.374	7.384–7.408
pCO_2_ [mmHg]	*69.6*	42.3	*45.1*	35–45
pO_2_ [mmHg]	*55.4*	38.5	35	35–45
HCO_3_^−^ [mmol/L]	*17.7*	27.8	26.4	21–53
Na^+^ [mmol/L]	*116.4*	*130.1*	138	135–148
K^+^ [mmol/L]	*2.96*	*2.51*	4.0	3.5–4.5
Cl^−^ [mmol/L]	*88.5*	100.5	102.2	98–107
Glucose [mmol/L]	*7.3*	*8.1*	4.0	4.1–5.9
Lactate [mmol/L]	*5.6*	1.1	1.2	1.0–1.7

*pCO*_*2*_ partial pressure of carbon dioxide; *pO*_*2*_ partial pressure of oxygen; *HCO*_*3*_^−^ bicarbonate ion; *Na* ^+^ sodium ion; *K* ^+^ potassium ion; *Cl* ^−^ chloride ion

^a^Reference ranges obtained with the iStat EG7+ device

Values in italic represent relevant modifications in the parameter

**Table 3:** Gelding serum biochemical parameters before and after anaphylactic reaction to sodium ceftriaxone, which occurred on day 1

	Basal	Day 6	Day 10	Day 20	Day 21	Day 28	Reference^a^
Direct bilirubin [mg/dL]	0.28	-	*1.10*	0.29	*0.35*	0.27	0.12–0.31
Indirect bilirubin [mg/dL]	0.06	-	0.40	*2.25*	*4.12*	0.51	0.07–1.07
Creatine phosphokinase [U/L]	146	-	*80*	*448*	*1162*	120	84–368
Urea [mg/dL]	15	21	26	26	20	41	14–41
Creatinine [mg/dL]	1.3	1.6	*0.9*	*0.9*	*0.7*	1.3	1.1–1.7
Aspartate Amino Transferase [U/L]	302	220	173	296	426	271	232–447
Gamma Glutamyl Transferase [U/L]	18	*68*	*21*	16	18	17	2.0–19.0
Total Protein [g/dL]	6.4	6.4	5.4	6.9	*7.6*	6.7	4.0–7.7
Albumin [g/dL]	2.4	-	2.9	2.1	2.3	1.7	1.7–3.2
Globulin [g/dL]	4.0	-	2.5	4.8	*5.3*	*5.0*	2.3–4.8
Fibrinogen [g/dL]	0.1	0.4	0.5	*1.2*	*0.9*	0.2	0.1–0.4

^a^References ranges obtained from the hematological analysis of 33 domestic horses of several breeds, serum negative for *Borrelia burgdorferi*, kept under the same management and feeding conditions

Values in italic represent relevant modifications in the parameter

### Case 2: Mare

**Fig. 3:**
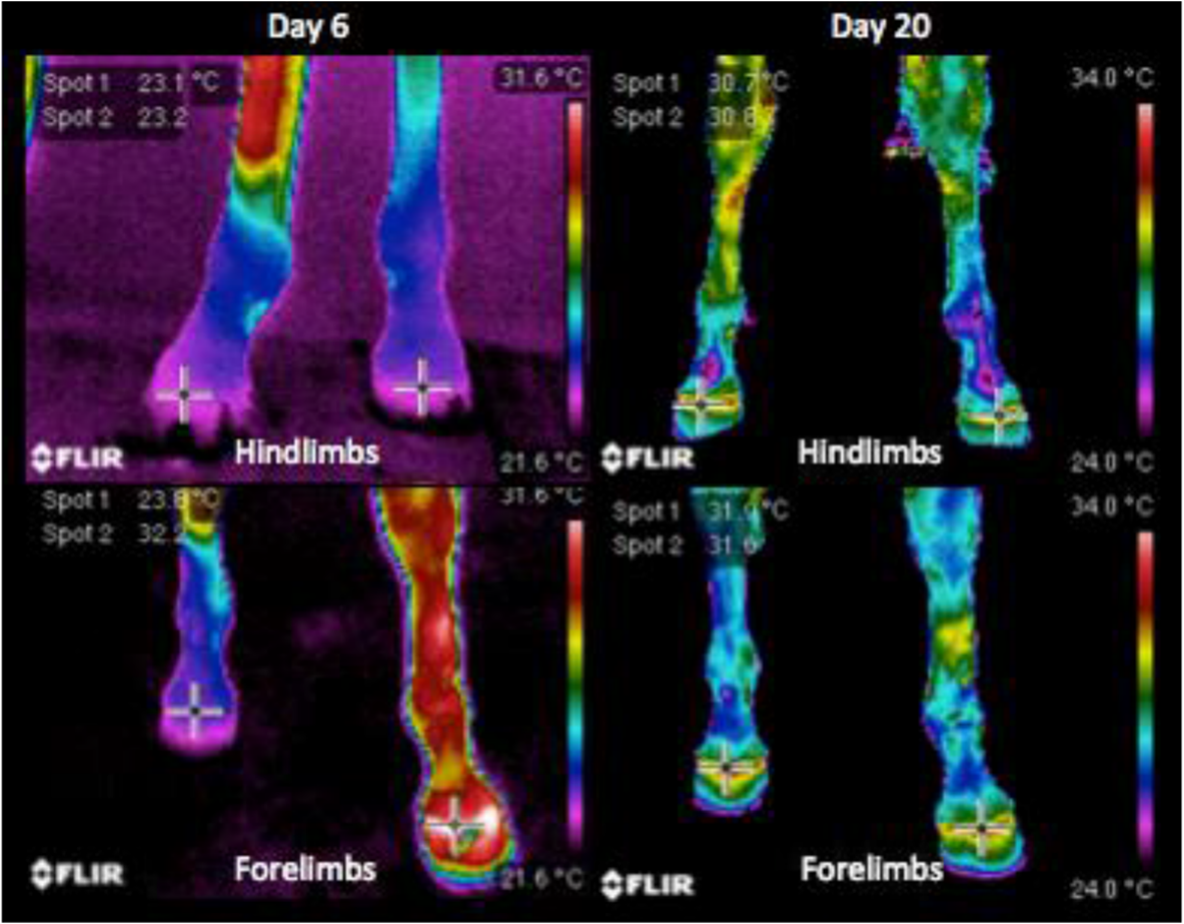
Infrared images to monitor the evolution of the laminitis treatment in the mare that had colic secondary to anaphylactic reactions to ceftriaxone sodium. Day 6 was the second day of temperature rise in the hoof. The crosses indicate the temperature of the hoof crown for each member. Temperature profile of the hooves was back to normal on Day 20. Spot 1: right limb, Spot 2: left limb

**Fig. 4:**
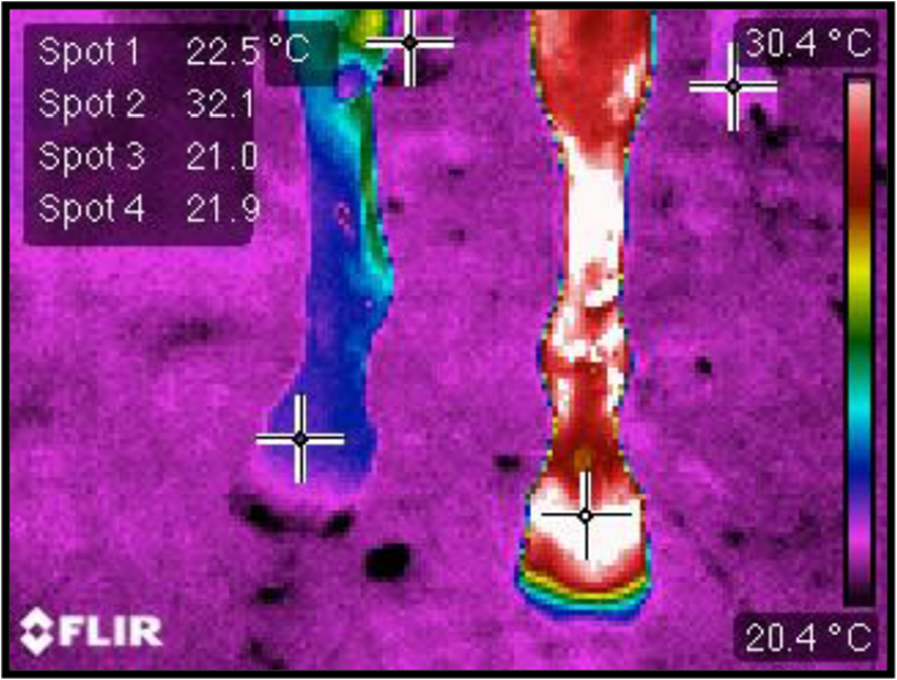
Infrared image of the four limbs of the mare 2 days after treatment started (Day 8). The crosses indicate the temperature of the hoof crown of each member. Spot 1: right forelimb, Spot 2: left forelimb, Spot 3: right hindlimb, Spot 4: left hindlimb

The Anglo-Arab mare weighing 420 kg, submitted to food fasting for 6 h, had a basal heart rate (HR) of 38 bpm, respiratory rate (RR) of 16 mrm, rosy mucous membranes, capillary refill time (CRT) of 2 s, rectal temperature (RT) 36.6 °C and hematological parameters within the reference range (Table 4, Basal) before starting the antibiotic treatment. After 95 mL of the ceftriaxone sodium (Day 1) solution had dripped, the mare presented paresis in the pelvic limbs, suggestive of circulatory hypotension. The antibiotic solution drip was suspended immediately, and within a few minutes, the mare was back to normal with no apparent need for pharmacological intervention. Two days after the anaphylactoid reaction (Day 3), the mare started showing signs of discomfort and slight abdominal distention. The physical examination revealed a HR of 52 bpm, RR of 34 mrm, RT 37.5 °C, CRT 3 s, pale mucosa and a severe intestinal hypomotility. No abnormalities were found upon rectal palpation, except for the presence of gas in the bowel loops and wall edema of the rectum. The colic event did not alter the hematological parameters or serum biochemistry of the patient (Tables 4 and 5, Day 3). The support treatment consisted of administering 3 L of 10 % DMSO solution, 10 ml intravenous dexamethasone, gastric lavage and100 mL of 30 % oral silicon solution to reduce bloating and flunixin meglumine (0.5 mg/kg), intravenously. The patient showed improvement in the intestinal motility 6 h after the drug treatment had started. Three days after the tympanic colic episode (Day 6), the temperature of the dorsal hoof wall of the left forelimb (LF) increased and the mare revealed strong pulse in the palmar digital arteries (Fig. 3, Day 6). Cryotherapy was performed in the region between the hoof and carpus for 1 h, three times a day, and intravenous flunixin meglumine, anti-endotoxemic dose (0.25 mg/kg), every 12 h. Two days after initiating the cryotherapy (Day 8), no improvement was noted in the LF temperature profile (Fig. 4). Therefore, along with cryotherapy, firocoxib was administered orally (0.1 mg/kg), every 24 h, in combination with pentoxifylline (8.4 mg/kg), every 12 h, for 10 days. At the end of the 10th day, the temperature profile of the left forelimb returned to normal values (Fig. 3, Day 20). The mare showed no signs of pain or lameness in the affected limb at any time; thus, suggesting the development of laminitis was treated within the prodromal phase. Therefore, no hoof radiographic assessment was performed. There were no alterations in the environment or feeding throughout ceftriaxone administration at the beginning of the colic period that would explain the abdominal discomfort occurrence and the resultant laminitis. Therefore, the authors suggest that the possible hypotension was associated with the liberation of inflammatory mediators which caused the subsequent laminitis. Table 6 summarizes the events that occurred with the two animals over time. Penicillins are the most important antibiotic class followed by cephalosporins, which contains a beta-lactam ring in their molecular structure. Both groups can induce hypersensitivity reactions mediated by IgE [18]. *In vitro* studies demonstrated that IgE antibodies are reactive to both terminals of cephalosporin molecules. This hybridoma formation has shown that cephalosporins can generate unique structures capable of inducing specific allergic reactions, which may exhibit cross-reactivity with penicillin in 5 to 15 % of the cases [19, 20]. In this study, both animals had been previously treated with penicillin without any signs of an allergic reaction, which suggests an anaphylactoid reaction. To date, there are no reports regarding the manifestation of hypersensitivity to cephalosporin in horses. This was one of the reasons why a drug of this group (ceftriaxone sodium) was chosen to treat the experimental infection with *Borrelia burgdorferi*. In addition to the cephalosporin pharmacokinetic studies conducted in horses [15–17], clinical trials were also conducted in camels [21], dogs [22], calves [23] and lactating goats [24] without any reports regarding the occurrence of hypersensitivity reactions. The evolution of the anaphylactoid symptoms in all of the tried horses was consistent with a severe hypersensitivity reaction in the gelding (without reaching shock parameters) and moderate reaction in the mare, which showed only signs of circulatory hypotension. Typically the symptoms of mild anaphylactoid reactions and hypersensitivity in horses are limited to local and dermatological [25, 26] changes. During a classical anaphylaxis, it is necessary a first substance exposure to immunologic sensitization and a cascade of events associated with the IgE molecule binding to basophils and mast cells occurs, triggering the release of histamine and other vasoactive substances [3, 27], such as serotonin, catecholamines, kinins, products of arachidonic acid and platelet activating factor [5]. The anaphylactoid reaction is a non-IgE mediated hypersensitivity response, caused by immune aggregates, complement activation, coagulation activation or autoimmune mechanisms, with the same clinical appearance of a classical anaphylaxis [27]. Both horses had never been exposed previously to sodium ceftriaxone and did not have any reaction to penicillin, resulting in an anaphylactoid reaction. Besides, it was not possible to know whether the massive death of spirochetes during antibiotic application also had correlation with clinical signs presented by both horses. Further evidences shall be collected in order to define if the anaphylactoid reaction was caused only by the sodium ceftriaxone or the interaction between the drug and the *B. burgdorferi* infection in horses. The pharmacological treatment with dexamethasone reversed the acute symptoms of the anaphylactoid reaction in the gelding, corroborating published studies [9, 28, 29]. It has been reported that anti-inflammatory non-steroidal drugs (NSAID’s) showed greater efficacy in reversing the cardiovascular and respiratory effects of experimentally induced anaphylaxis in horses compared to antihistamines. Together with corticoids, the animals may also have benefited from epinephrine for controlling acute signs of hypotension and edema [25]. The vascular and hemodynamic changes that occurred during reaction in the gelding resulted in a mural edema of various intestinal segments, in addition to *ileus*, with signs of abdominal discomfort. During celiotomy, mural edemas were observed on the colon and small intestine, as well as a large amount of liquid and gaseous content and petechiations promoted by fragility and alteration of vascular permeability. While recovering from the surgical procedure, the gelding erythrogram displayed parameters on the lower limit of normality, neutrophilia with a regenerative left shift or leukopenia (Day 3), which may have been caused by the steroid therapy or anaphylactoid reaction. Along the scenario of metabolic acidosis, there were also electrolyte abnormalities on the day following the anaphylactoid reaction, with decreased plasma concentrations of sodium, potassium and chlorine. The anaphylaxis report where a reaction was induced by intravenous administration of breast milk on a foal lists the same pattern of changes on the following day [11]. Such changes can be directly associated to the systemic inflammatory response syndrome induced by the beta lactam compound [30]. During an anaphylactoid reaction, large amounts of fluids are sequestered to tissue compartments richer in mast cells and basophils due to antigen-antibody bonds that promote the release of vasoactive amines and hence induce the local vasodilatation. It is known that tissue concentrations (muscle and viscera) of sodium, potassium and chloride increase during anaphylaxis, resulting in a homeostatic imbalance of these serum electrolytes [31]. A significant increase in the concentrations of plasma glucose and lactate was also observed, which may be linked to an increased anaerobic metabolism due to difficult tissue gas exchange resulting from the edema. This fact can be explained by the concomitant increase of venous partial pressures of oxygen and carbon dioxide, shown a day after the anaphylactoid reaction in the gelding. Hematologic and metabolic changes may have predisposed the gelding to the appearance of Theileriosis on Day 18. However, the enzymatic biochemical profile of the gelding did not change significantly as a result of anaphylaxis but only after the hemoparasitosis. In the mare, it could be that the anaphylactoid mediators caused the possible hypotension and this may be responsible for starting the systemic inflammatory response that resulted in abdominal discomfort and laminitis (day 6). The inflammatory response was detected by the 10th day, through laboratory confirmation of neutrophilia and increased plasma fibrinogen. The infrared images show apparent temperature increase in the hoof of the left forelimb (day 6), which responded adequately to the non-steroidal anti-inflammatory therapy, cryotherapy and peripheral vasodilator, returning to normal on day 20 post infection.

**Table 4:** Mare red blood cell count before and after anaphylactic reaction to sodium ceftriaxone, which occurred on day 1

	Basal	Day 3	Day 10	Day 28	Reference^a^
Erythrocytes x 10^6^ [cells/ml]	8.3	7.1	8.4	7.4	6.0–9.7
Hemoglobin [g/dL]	13	11.2	13	11.5	8.3–14.4
Hematocrit [%]	39	31	39	35.0	30–44
MCV [fL]	47	-	46.4	47.3	36–52.1
MCH [pg]	15.7	-	15.5	15.5	11.5–18.2
MCHC [g/dL]	33.3	-	33.3	32.9	31.2–34.9
Leucocytes [cells/uL]	8600	6600	10000	6900	6400–10600
Basophiles [cells/uL]	0	0	0	0	0
Eosinophils [cells/uL]	*344*	66	200	138	0–320
Segmented Neutrophils [cells/uL]	4902	4224	7300	3588	2775–7530
Rod Neutrophils [cells/uL]	258	0	*800*	207	94–420
Lymphocytes [cells/uL]	2924	2244	1500	2898	1088–5096
Monocytes [cells/uL]	172	66	200	69	90–318
Platelets [cells/uL]	151000	154000	191000	165000	90000–322000

^a^Reference ranges obtained for total blood cell count for 33 domestic horses of different breeds, serum negative for *Borrelia burgdorferi*, kept under the same feeding and management conditions

Values in italic represent relevant modifications in the parameter

**Table 5:** Mare serum biochemical parameters before and after anaphylactic reaction to sodium ceftriaxone, which occurred on day 1

	Basal	Day 3	Day 10	Day 28	Reference^a^
Direct bilirubin [mg/dL]	0.13	0.39	*1.1*	0.18	0.12–0.31
Indirect bilirubin [mg/dL]	0.10	*1.66*	0.2	0.63	0.07–1.07
Creatine phosphokinase [U/L]	166	–	276	223	84–368
Urea [mg/dL]	13	25	18	35	14–41
Creatinine [mg/dL]	1.1	1.6	1.1	1.0	1.1–1.7
Aspartate Amino Transferase [U/L]	424	199	263	368	232–447
Gamma Glutamyl Transferase [U/L]	*33*	*76*	*36*	*23*	2.0–19.0
Total Protein [g/dL]	6.5	6.4	6.6	7.2	4.0–7.7
Albumin [g/dL]	2.4	2.44	3.4	2.3	1.7–3.2
Globulin [g/dL]	4.1	-	3.2	*4.9*	2.3–4.8
Fibrinogen [g/dL]	0.1	0.5	*0.9*	0.2	0.1–0.4

^a^References ranges obtained from the hematological analysis of 33 domestic horses of several breeds, serum negative for *Borrelia burgdorferi*, kept under the same management and feeding conditions

Values in italic represent relevant modifications in the parameter

**Table 6:** Timetable of the events described for the anaphylactic reaction to sodium ceftriaxone of a horse and a mare

Chronology	Gelding	Mare
Basal	Total blood cell count before treatment started	Total blood cell count before treatment started
Day 1	Administration of ceftriaxone sodium and anaphylaxis	Administration of ceftriaxone sodium and anaphylaxis
Day 2	Tympanic colic and laparotomy	Stable
Day 3	Stable	Tympanic colic resolved clinically
Day 6	Stable	Prodromal laminitis and treatment
Day 8	Stable	Change in the laminitis treatment
Day 18	Theileriosis treated with imidocarb	Stable
Day 20	6 L of whole blood transfusion	Stabilization of the clinical symptoms
Day 21	Gastritis	Stable
Day 28	Clinical symptoms back to normal	Stable

## Conclusions

An anaphylactoid reaction is a potential risk to the lives of horses. It is characterized by hypotension and potential cardiovascular collapse, associated with redistribution of the blood volume in the lung and gastrointestinal tract, and may progress to tissue edema and hypertension. Horses that survive these reactions may develop a number of complications, including colic syndrome, laminitis and manifestation of latent infectious diseases.

## Abbreviations

C3A: Complement componnet 3A
C567: Complement components 5, 6 and 7
C5A: Complement component 5A
CEUA: Comitê de Ética e Uso Animal
CRT: Capilary refil time
dL: Deciliter
DMSO: Dimetil sulfoxide
FAPESP: Fundação de Ampoaro e Apoi à Pesquisa do Estado de SP
g: Gram
HR: Heart hate
IgE: Imunoglobulin E
kg: Kilogram
L: Liter
LF: Left forelimb
LH: Left hindlimb
MCH: Mean corpuscular hemoglobin
MCHC: Mean corpuscular hemoglobin concentration
MCV: Mean corpuscular volume
mg: Milligram
MG: Minas Gerais
NSAID: Non steroidal anti-inflamatory drugs
PCR: Polymerase Chain Reaction
RF: Right forelimb
RH: Right hindlimb
RJ: Rio de Janeiro
RR: Respiratory rate
RT: Retal temperature
s: Second
SP: São Paulo
U: Units
UNESP: Universidade Estadual Paulista

## Footnotes

1 Celltriaxon®, Agila Especialidades Farmacêuticas, Campos dos Goytacazes – RJ – Brazil. Batch: 7402209. Manufacture: 10/2013. Validity: 10/2015.

2 Angiocath® 14G, BD, São Paulo – Brazil.

3 Novapen® – Hertape Calier, Juatuba – MG.

4 Imizol® – MSD Saúde Animal, São Paulo – SP.

5 Gastrozol® 2.28g – Hertape Calier, Juatuba – MG.
